# YouTube Educational Videos for Peripheral Block Learning and Application: A Survey of Turkish Anesthesiologists; How Successful

**DOI:** 10.5152/eurasianjmed.2023.23075

**Published:** 2023-10-01

**Authors:** Selçuk Alver, Bahadir Ciftci, Birzat Emre Gölboyu, Mohammad Sargolzaeimoghaddam, Cem Erdoğan, Haci Ahmet Alıcı, Ali Ahiskalioglu

**Affiliations:** 1 Department of Anesthesiology and Reanimation, Istanbul Medipol University Faculty of Medicine, Istanbul, Turkey; 2 Department of Anesthesiology and Reanimation, Katip Çelebi University Faculty of Medicine, İzmir, Turkey; 3 Medical Student, Istanbul Medipol University Faculty of Medicine, Istanbul, Turkey; 4 Department of Algology, Istanbul Medipol University Faculty of Medicine, Istanbul, Turkey; 5 Department of Anesthesiology and Reanimation, Ataturk University School of Medicine, Erzurum, Turkey.

**Keywords:** Regional anesthesia, nerve block, ultrasound, YouTube, survey

## Abstract

**Objective:**

The aims of this survey study were to evaluate the contribution of YouTube to nerve-block learning/education and the advantages and disadvantages of the YouTube.

**Materials and Methods:**

A total of 24 questions were selected for the survey by consensus of the authors. Information in the form of web data was obtained through an electronic data form that was distributed via WhatsApp to known email addresses and phone numbers of 300 practitioners (anesthesia residents, anesthesiologists, and academicians). There were a total of 24 questions on the survey. The first section included 5 questions collecting demographic data, and the second part encompassed 19 questions about the YouTube nerve block videos.

**Results:**

Among the participants, 232 of practitioners (86.9%) performed peripheral nerve blocks, and only 35 practitioners (13.1%) had no experience of nerve blocks so and used YouTube videos for educational purposes. According to our results, YouTube videos frequently improved performance. In addition, YouTube improved the training of practitioners in terms of the type of block procedure, identifying anatomical landmarks, target structures like nerves and blood vessels, needle visualization, needle depth, and patient position.

**Conclusion:**

YouTube contributes to the performance of regional anesthesia and to learning at all academic levels. It should not be forgotten that such videos are not peer reviewed by professionals in the relevant field.

**Main Points**

- YouTube is a popular social media platform nowadays.
- Regional anesthesia field is a popular area in anesthesia practice.
- Clinicians can watch the videos about regional anesthesia in YouTube.
- According to our results, YouTube contribute to the regional anesthesia learning.

## Introduction

Peripheral nerve blocks are widely used in our daily anesthesia practice for the guidance of ultrasound (US). Anatomical landmarks, such as vascular structures, bones, and nerves, are easily seen under the US. Using the US for guidance increases the success and efficacy of nerve blocks, and the rate of complications is lower than with landmark-guided techniques.^1^ Because we can see tissues in real time, it is simple and safe to perform US-guided techniques. While the experienced/expert anesthesiologist can visualize the anatomical structures on the US, residents and less-experienced anesthesiologists may have difficulties while performing the block procedure.^2^ Clinicians typically use internet videos for information about techniques before a procedure. Therefore, video-sharing platforms have become quite popular among health professionals as a source of medical knowledge and as educational tools.^3-9^ However, videos and information available on the internet may not be useful and do not always present accurate knowledge about the related topic. YouTube (www.youtube.com) is a popular video-sharing platform that is commonly used as a video website.^5,9^ Several videos of medical procedures, such as regional anesthesia, are available on YouTube.^3-6^ Some of the videos have been prepared as visual educational guides.^4,5^ However, a number of studies have shown that the reliability, understandability, and compliance of many such medical videos are lacking or they are not approved by professionals in the respective fields.^3-7^

In the literature, there are studies that evaluate YouTube videos regarding neuraxial blocks and lumbar puncture techniques.^3^ In our study, we evaluated the efficacy of YouTube videos with US-guided regional anesthesia techniques among anesthetists in Turkey. We evaluated which regional techniques are used most frequently and the advantages and disadvantages of YouTube contributions to the block technique. The aims of this study were to evaluate (1) the contribution of YouTube to nerve block learning and education and (2) the advantages and disadvantages of YouTube.

## Materials and Methods

Our research was approved by the Ethics and Research Committee of Istanbul Medipol University for a survey study conducted between November 2022 and February 2023. After obtaining ethical approval, the survey questions were prepared according to particular YouTube videos regarding current nerve-block procedures that practitioners frequently watch and use as guidance. We prepared the questions by taking samples from previous works.^3,4,6^

### Data Collection

A total of 24 questions were selected for the survey by consensus of the authors. Information in the form of web data was obtained through an electronic data form that was distributed via WhatsApp to known email addresses and phone numbers of 300 practitioners (anesthesia residents, anesthesiologists, and academicians). The confidentiality of participants’ personal data was maintained by ensuring the anonymity of the phone sending the link to a completed form. Before starting the survey, participants were informed that their personal data would be protected. The replies to the surveys were checked for possible resubmissions. The survey did not include any questions related to age group or educational status. Practitioners who applied regional techniques were asked to detail their work schedules and which departments they most frequently worked with for anesthesia. The participants were asked to detail at which stage of the process they watched YouTube videos, what the advantages and disadvantages of such videos were, and which procedures they watched most frequently. Practitioners were also asked to detail the contributions of videos they watched to their performance. In this manner, we were able to determine which stages and how often YouTube videos demonstrating regional techniques are used in anesthesia practice and the advantages and disadvantages of the videos for training.

### Creation and Content of the Survey

There were a total of 24 questions on the survey.

**Part** **1:** The first section included 5 questions collecting demographic data (Table 1).

**Part 2:** The second part encompassed 19 questions about the YouTube nerve-block videos (Table 2).

### Statistical Analyses

The pooled data were evaluated using the Statistical Package for Social Sciences (SPSS) 22.0 statistical program (IBM SPSS Corp.; Armonk, NY, USA). Frequency distributions were calculated and presented as number and percentage. Pearson’s chi-square test and Kruskal–Wallis H test were used to compare categorical data between the groups.

## Results

Our survey was sent to anesthetists working in Turkey via telephone or e-mail, 300 practitioners completed the survey. The responses received within 15 days were included in the study. Because it was determined that 33 practitioners had not completed the questionnaire, 267 answers were considered to be valid responses. There were 137 female (51.3%) and 130 male participants. The age range of 36.7% of the participants was 25-30 years old, making up majority of the participants in our survey. Among the practitioners, 6 were professors (2.2%), 14 were associate professors (5.2%), 18 were physicians (6.7%), 106 were specialist doctors (39.7%), and 123 were resident doctors (46.1%). When the academic level achieved was lower, the rate of YouTube usage increased. A majority of the participants, 91.8%, worked in operating rooms (Table 2).

Among the participants, 232 of practitioners (86.9%) performed peripheral nerve blocks, and only 35 practitioners (13.1%) had no experience of nerve blocks and so used YouTube videos for educational purposes. According to our results, YouTube videos frequently improved performance. In addition, YouTube improved the training of practitioners in terms of the type of block procedure, identifying anatomical landmarks, target structures like nerves and blood vessels, needle visualization, needle depth, and patient position.

Conversely, YouTube did not improve knowledge about dermatomes, the choice of an appropriate local anesthetic solution, an appropriate US probe and frequency, and in-plane or out-of-plane approaches (Table 2).

**Figure 1. A-F.**
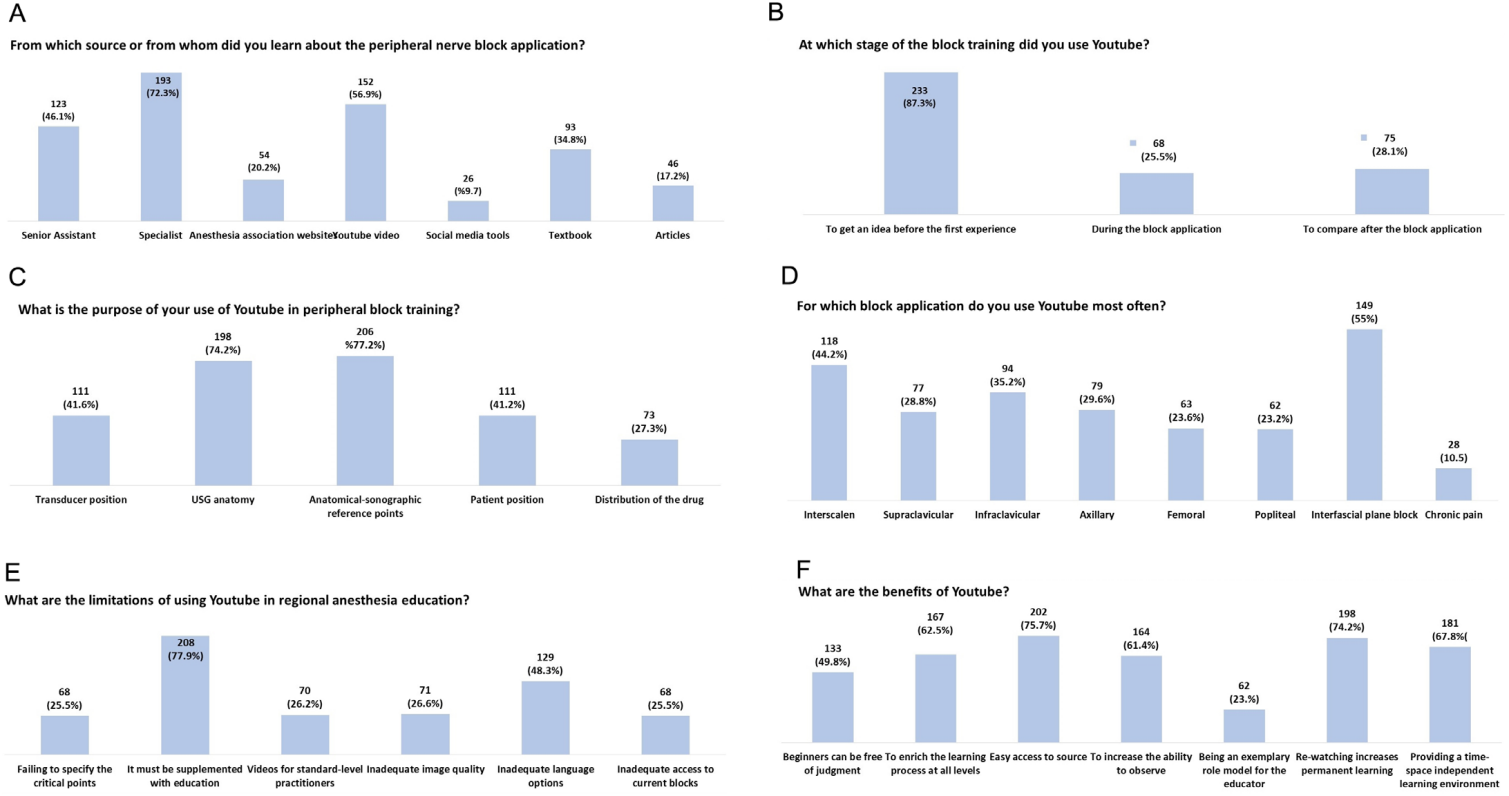
The statistical analyses of the survey answers

Practitioners were asked, “Where did you learn the peripheral nerve block application?” based on their asnwers 193 learned from specialist anesthesiologists, 152 from YouTube videos, 123 from senior residents, 93 from related books, 54 from high-quality anesthesia journals, 46 from national or international articles, and 26 from social media tools. According to our results, YouTube has an important role in peripheral nerve block learning and training (Figure 1A).

Practitioners answered the question, “At what stage of your nerve block training did you use the YouTube app?” based on their answers 233 participants (87.3%) watched to get an idea of the procedure before their first experience, 68 (25.5%) watched during the block procedure, and 75 (28.1%) watched after performing a block procedure to compare with the experience. YouTube is usually preferred before the first block is performed. Experienced practitioners also preferred YouTube during and after the procedure (Figure 1B).

Practitioners stated that the YouTube application contributed most to learning about sonographic anatomy and reference landmarks (Figure 1C).

The question, “For which block do you use YouTube the most?” revealed that 149 practitioners choose interfacial plane blocks. This result indicates that new methods increase interest in YouTube applications, and the application improves knowledge and innovation among practitioners (Figure 1D).

With regard to a question about the disadvantages of YouTube use in regional anesthesia training, most of the practitioners (n = 208, 77.9%) stated that the application alone is insufficient. Other disadvantages listed included a lack of language options in the videos, the fact that critical landmarks are not specified in some videos, standard and classical videos, and the lack of the knowledge about current blocks (Figure 1E).

The majority of the practitioners (n = 202, 75.7%) emphasized the importance of easy access to the source when asked about the advantages of using YouTube in performing a block. A total of 198 practitioners stated that rewatching videos increased learning, while 181 indicated that YouTube provides time and place independent conditions, and 167 practitioners stated that it enriched the learning process at all levels. In addition, 164 practitioners stated that it increased their observational ability, and 133 stated that an advantage of YouTube was that it shortened the time they spent at the beginner level (Figure 1F).

## Discussion

According to the results of our survey study, YouTube contributes to the performance and learning of regional anesthesia at all academic levels. According to our results, YouTube videos frequently improved performance. YouTube improved the training of practitioners in terms of the type of block application (identifying anatomical landmarks, needle depth, and patient position). However, YouTube did not improve knowledge about dermatomal coverages, local anesthetic solutions, and in-plane/out-of-plane approaches. YouTube application improves knowledge and innovation among practitioners.

The US-guided regional anesthesia is a popular and developing topic in anesthesia, and novel techniques have been described in the literature.^10-14^ Because the US-guided techniques are simple, safe, and have opioid-sparing effects, they are an emerging topic in daily anesthesia practice. Video-sharing platforms are important for learning and performing these techniques; however, it is important to keep in mind that they do not undergo peer review.^5^ Survey studies are important for sharing knowledge about up-to-date techniques among clinicians.^5-15^ Our results show that YouTube improves knowledge about regional anesthesia and contributes to education due to the advantages of this source, such as easy access to the resource, increased ability to observe, and the ability to re-watch enhancing permanent learning. The recent increase in the use of interfacial plane blocks and their frequent viewing on YouTube is an important evidence that the application is open to innovation and contributes to education.^15-17^ In addition, in our study, the use of YouTube by practitioners at all academic levels, from professor to resident anesthetist, is considered a result of YouTube’s advantages in performing block procedures. According to our results, YouTube is commonly used by anesthesia residents in the operating room. Given the popularity of YouTube and the number of views of related videos, practitioners apparently seek audiovisual information from these platforms. An important step in resident skills development involves observing procedures on models or patients before performing it and after the skill has been learned from a textbook or through a professional health-care instructor.^18^

In contrast, according to our results, YouTube alone is insufficient and should be supported with education because of the disadvantages of YouTube, which include factors such as lack of video quality, lack of language options, difficulty in accessing up-to-date techniques, and lack of explanations of critical landmarks. Incorrect information about this topic can lead to complications such as hematoma, pneumothorax, and neural injury. Unfortunately, it has been demonstrated that most of these videos have not been reviewed by professionals in the field.^5^

Tewfik et al^4^ compared high-quality educational YouTube videos with anesthesia websites (NYSORA, ACEP, and USRA). While the YouTube videos may be used to learn or demonstrate nerve blocks, Tewfik et al recommended using only high-quality sources. In our survey study, video quality was not evaluated, and the questionnaire was prepared considering all available block videos on YouTube. Therefore, a disadvantage of our study is that YouTube alone is insufficient and must be supported by education and real-time training on patients. Selvi et al^6^ evaluated the content of material on YouTube relevant to brachial plexus block procedures and the quality of videos as a source of digital visual information. In their study, the YouTube search was performed using keywords associated with brachial plexus block, and out of 374 videos, 86 were included. The researchers classified the videos separately according to the questionnaires used, in which Questionnaire 1 (Q1) was prepared according to the ASRA/Miller’s anesthesia guidelines as a reference book and Questionnaire 2 (Q2) was formulated using a modification of the criteria in *Guidelines Assessment for Video Media*. In total, 72 videos regarding the US-guided blocks and 14 videos on nerve stimulator blocks were reviewed. In their conclusion, they reported that most of the videos examined for their study lacked the comprehensive approach needed to safely guide people seeking information about brachial plexus block. Tulgar et al^3^ evaluated YouTube as a source of neuraxial anesthesia techniques, considering 40 videos after exclusions. They reported that videos prepared by institutes and societies had better educational value. Videos should be designed in adherence with available up-to-date guidelines considering appropriate step-by-step explanations of each procedure. De Cassai et al^5^ evaluated the educational value and quality of 21 YouTube videos about the erector spinae plane block. They compared the difference in quality between academic and non-academic videos and reported that clinicians should be extremely cautious about using video-sharing platforms due to the limited technical and video quality on YouTube.

### Limitations

Our study had some limitations. We included all videos about regional anesthesia on YouTube and did not apply any exclusion criteria. All anesthesia practitioners from all academic levels, resident to professor, took part in the survey. We did not specify a type of regional anesthesia technique, such as spinal, epidural, nerve block, or interfacial plane block. Further studies with larger sample sizes are needed.

## Conclusion

YouTube contributes to the performance of regional anesthesia and to learning at all academic levels. However, it should not be forgotten that such videos are not peer reviewed by professionals in the relevant field.

## Figures and Tables

**Table 1.** The Demographic Part of Survey (Part 1)

	n = 267 (%)
Gender	
Female/male	130 (51.3%)/137 (48.7%)
Age (year)	
25-30	98 (36.7%)
31-35	73 (27.3%)
36-40	42 (15.7%)
>41	54 (20.2%)
Title	
Resident	123 (46.1%)
Specialist	106 (39.7%)
Assistant professor	18 (6.7%)
Associate professor	14 (5.2%)
Professor	6 (2.2%)
Experience (year)	
<5	109 (40.8%)
6-10	66 (24.7%)
11-15	42 (15.7%)
16-20	29 (10.9%)
>21	21 (7.9%)
Department	
Operating room	245 (91.8%)
Intensive care	20 (7.5%)
Pain medicine	2 (0.7%)

**Table 2.** The Survey Questions of the Study (Part 2)

n = 267	Yes	No
Do you perform peripheral nerve blocks?	232 (86.9%)	35 (13.1%)
YouTube videos influenced the block’s performance?	249 (93.3%)	18 (6.7%)
YouTube videos explain in which situations the block can be used?	195 (73%)	72 (27%)
Are anatomical landmarks clearly indicated in YouTube videos?	238 (89.1%)	29 (10.9%)
Target dermatomes innervated by the nerves explained in the YouTube videos?	115 (43.1%)	152 (56.9%)
Vascular and nerve structures related to the target nerve mentioned in YouTube videos?	182 (68.2%)	85 (31.8%)
Information about local anesthetics clearly explained in YouTube videos?	61 (22.8%)	206 (77.2%)
The sonoanatomical images and anatomical structures easy to detect in YouTube videos?	216 (80.9%)	51 (19.1%)
The needle’s ultrasound image quality satisfactory in the YouTube video?	233 (87.3%)	34 (12.7%)
The instructions for depth movements, alignment, and needle direction clearly explained in the YouTube videos?	149 (55.8%)	118 (44.2%)
The technical information about the probe selection and frequency of the ultrasound device explained in the YouTube videos?	92 (34.5%)	175 (65.5%)
Information about the in-plane or out-of-plan etechniques presented in the video?	102 (38.2%)	165 (61.8%)
Patient position information clearly stated in the YouTube videos?	196 (73.4%)	71 (26.6%)
